# Matrix Resistance Toward Proteolytic Cleavage Controls Contractility‐Dependent Migration Modes During Angiogenic Sprouting

**DOI:** 10.1002/advs.202305947

**Published:** 2024-03-13

**Authors:** Martin S. Weiß, Giuseppe Trapani, Hongyan Long, Britta Trappmann

**Affiliations:** ^1^ Bioactive Materials Laboratory Max Planck Institute for Molecular Biomedicine Röntgenstraße 20 48149 Münster Germany; ^2^ Department of Chemistry and Chemical Biology TU Dortmund University Otto‐Hahn‐Straße 6 44227 Dortmund Germany

**Keywords:** angiogenesis, cell migration, cell‐ECM interactions, organ‐on‐chip, synthetic hydrogels

## Abstract

Tissue homeostasis and disease states rely on the formation of new blood vessels through angiogenic sprouting, which is tightly regulated by the properties of the surrounding extracellular matrix. While physical cues, such as matrix stiffness or degradability, have evolved as major regulators of cell function in tissue microenvironments, it remains unknown whether and how physical cues regulate endothelial cell migration during angiogenesis. To investigate this, a biomimetic model of angiogenic sprouting inside a tunable synthetic hydrogel is created. It is shown that endothelial cells sense the resistance of the surrounding matrix toward proteolytic cleavage and respond by adjusting their migration phenotype. The resistance cells encounter is impacted by the number of covalent matrix crosslinks, crosslink degradability, and the proteolytic activity of cells. When matrix resistance is high, cells switch from a collective to an actomyosin contractility‐dependent single cellular migration mode. This switch in collectivity is accompanied by a major reorganization of the actin cytoskeleton, where stress fibers are no longer visible, and F‐actin aggregates in large punctate clusters. Matrix resistance is identified as a previously unknown regulator of angiogenic sprouting and, thus, provides a mechanism by which the physical properties of the matrix impact cell migration modes through cytoskeletal remodeling.

## Graphical abstract

Endothelial cell migration through the extracellular matrix is a crucial step during angiogenic sprouting. However, the impact of physical matrix properties on cell behavior in 3D is unclear. In this study, matrix resistance toward proteolytic cleavage is defined as a previously unknown microenvironmental cue that toggles endothelial cells between collective and single‐cell migration modes.

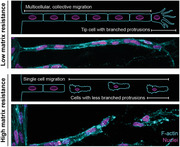

## 1 Introduction

One process of fundamental importance for embryonic development and the progression of many diseases, such as cancer, is sprouting angiogenesis, the formation of new blood vessels from preexisting vasculature.^[^ ^1^ ^]^ This complex, multistep process is initiated by endothelial cells (ECs) exiting a parent blood vessel to migrate through the surrounding 3D extracellular matrix (ECM), a key regulator of cell function.^[^ ^2^ ^]^ While biochemical ECM signals activating cellular integrins are well‐characterized regulators of angiogenesis,^[^ ^3^ ^]^ the role of physical ECM properties, which have recently been established as functionally important cues in 3D tissue microenvironments,^[^ ^4^ ^]^ remains elusive. Some studies have shown that changes in ECM confinement, which restricts cellular motility, can induce a phenotypic switch from single‐cell to collective migration in cancer cells^[^ ^5^ ^]^; while ECs are also able to alter between individual and multicellular migration patterns,^[^ ^6^ ^]^ it is unknown whether physical ECM properties are the regulators of this switch.

In order to understand whether and how the physical properties of the surrounding tissue microenvironment regulate EC migration, 3D model systems that recapitulate the structural features of native angiogenesis in an environment that allows for full and independent control over ECM properties are needed. Suitable tools to address such questions include recently developed microfluidic devices in which chemokine gradients trigger ECs to sprout from microchannels into a surrounding hydrogel.^[^ ^7^ ^]^ However, most of these models incorporate 3D matrices of natural origin, such as fibrous collagen^[^ ^7^, ^8^ ^]^ or fibrin.^[^ ^9^ ^]^ Due to their complex nature, it is very difficult to tune native ECM parameters independently, making it difficult to attribute the observed cellular phenotypes to specific matrix properties.^[^ ^10^ ^]^

To overcome this limitation, we and others have developed synthetic hydrogels based on a protein‐ and cell‐inert background onto which biochemical and mechanical cues of interest can be added one by one.^[^ ^11^ ^]^ By integrating such a hydrogel into a microfluidic device that mimics natural angiogenesis, our group recently uncovered an important microenvironmental cue that regulates the multicellularity of angiogenic sprouts: matrix crosslinking.^[^ ^7d^ ^]^ Specifically, we found that lightly crosslinked matrices that were easily degraded by cells triggered fast EC migration and, as a consequence, disengagement of cell–cell contacts. However, the degree of matrix crosslinking not only changes the matrix's degradability but also its stiffness, which is a major regulator of cellular mechanotransduction^[^ ^12^ ^]^; it remains to be understood whether and how matrix stiffness impacts EC migration phenotypes during angiogenesis.

Here, we demonstrate that highly crosslinked, stiff matrices, which are resistant toward proteolytic cleavage by cells, induce ECs to switch from a collective to an alternative, single‐cell migration strategy to physically navigate through their environments. Cells adapt to this microenvironmental change by upregulating cellular contractility, which is accompanied by cytoskeletal re‐organization, loss of polarity markers, and reduced branched protrusions.

## 2 Results

**Figure 1:**
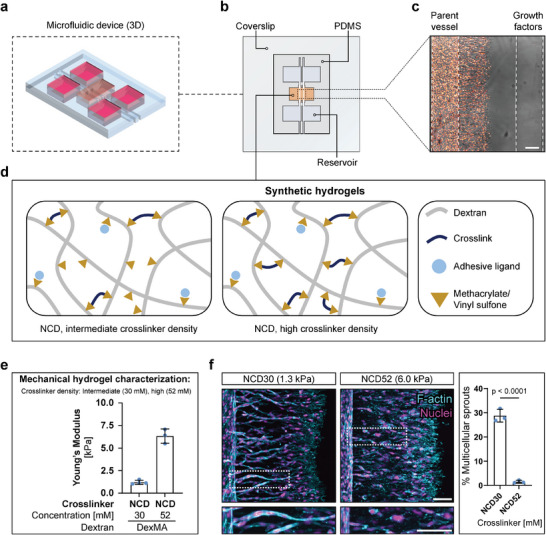
Matrix crosslinking regulates angiogenic sprout multicellularity. a) 3D schematic of the in vitro angiogenesis model. b) The microfluidic platform is based on a PDMS housing, and integrates a dextran‐based synthetic hydrogel with two embedded, parallel channels, each connecting to independent fluid reservoirs. One channel is lined with HUVECs, mimicking a parent blood vessel, the other channel serves as a source for pro‐angiogenic chemokines, creating a gradient toward the cell channel. c) Brightfield image of HUVECs invading a dextran hydrogel (nuclei in orange, scale bar = 200 µm). d) Design of dextran‐based hydrogels functionalized with cell adhesive peptides and crosslinked with tunable concentrations of NCD peptide. e) Young's modulus of DexMA hydrogels crosslinked with intermediate (30 × 10^−3^ m) and high (52 × 10^−3^ m) concentrations of NCD peptide. f) Composite fluorescence images of 3D projections showing F‐actin (cyan) and nuclei (magenta) of migrating HUVECs in DexMA hydrogels crosslinked with varying concentrations of NCD peptide, fixed after 3 and 10 days of migration (at similar invasion depths), respectively. Quantification of sprout multicellularity: Percentage of nuclei in collective (six or more nuclei) sprouts connected to the parent vessel, relative to the total number of nuclei inside the hydrogel (*n* = 3 independent experiments, scale bar = 100 µm). All data are presented as a mean ± s.d., statistical significance was determined from a *p* < 0.05 (two‐tailed unpaired Student's *t*‐test).

To investigate how matrix stiffness regulates angiogenic sprouting, we employed our previously established biomimetic model, which recapitulates the most important steps of natural blood vessel formation in a controlled in vitro environment. This model incorporates a synthetic hydrogel with tunable stiffness inside a microfluidic device that also captures the structural features of native angiogenic sprouting (**Figure** **1**a,b). Specifically, human umbilical cord vein endothelial cells (HUVECs) lining a parent channel are induced to sprout into the surrounding hydrogel by a chemokine gradient formed through the addition of a titrated pro‐angiogenic cocktail to a second, parallel source channel (Figure 1c). The hydrogel is based on dextran functionalized with methacrylates (DexMA) or vinyl sulfones (DexVS) as a protein‐ and cell‐inert backbone, to which cysteine‐containing cell‐adhesive peptides can be coupled through Michael‐type addition. Crosslinking through dicysteine‐functionalized matrix metalloproteinase (MMP)‐sensitive peptides taken from the natural cleavage site of collagen type I (termed NCD for “native collagen degradability”) renders the hydrogel suitable for the cell cleavage required for 3D cell spreading and migration (Figure 1d). Tuning the crosslinker concentration gives access to hydrogels of different stiffnesses (Figure 1e).

We first used this system to study how matrix stiffness impacts EC migration during angiogenic sprouting by changing the density of MMP labile crosslinks (Figure S1, Supporting Information). Since migrating cells have to cleave more crosslinks in stiffer matrices, resulting in slower migration compared to soft hydrogels, we fixed samples at different time points when the cells and sprouts reached the same invasion depth. In line with our previous observations,^[^ ^7d^ ^]^ we found that cells migrated as multicellular strands through intermediately crosslinked hydrogels (1.3 kPa), whereas highly crosslinked environments (6.0 kPa) only supported scattered, single‐cell migration (Figure 1f). These migration mode changes were not due to variations in cell proliferation (Figure S2, Supporting Information) or chemokine gradient profiles (Figure S3, Supporting Information), potentially resulting from differences in culture periods between the two crosslinking conditions. While we previously determined differences in matrix invasion speed to be the main regulator of the migration mode switch when going from low crosslinking densities (single‐cell migration) to intermediate crosslinking densities (collective migration),^[^ ^7d^ ^]^ it remained unclear why highly crosslinked matrices, in which invasion is slow, also only support single‐cell migration.

**Figure 2:**
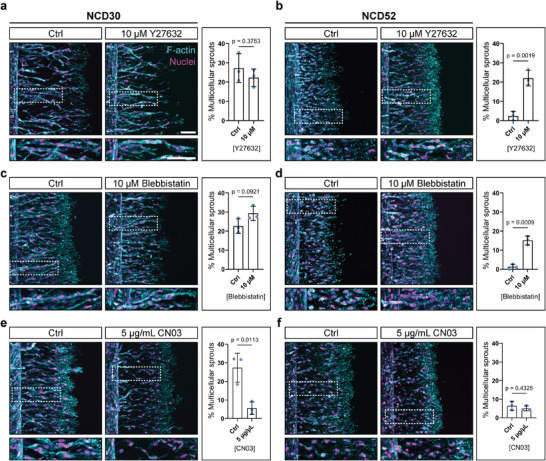
RhoA‐ROCK‐myosin II signaling regulates endothelial sprout multicellularity in hydrogels of different crosslink density. a, b) HUVECs invading DexMA hydrogels crosslinked with 30 × 10^−3^ m (a) and 52 × 10^−3^ m (b) NCD peptide, fixed after 3 and 10 days of migration (at similar invasion depths), respectively, with and without treatment of ROCK inhibitor Y27632. c, d) HUVECs invading DexMA hydrogels crosslinked with 30 × 10^−3^ m (c) and 52 × 10^−3^ m (d) NCD peptide, fixed after 3 and 10 days of migration, respectively, with and without treatment of myosin II inhibitor blebbistatin. e, f) HUVECs invading DexMA hydrogels crosslinked with 30 × 10^−3^ m (e) and 52 × 10^−3^ m (f) NCD peptide, fixed after 3 and 10 days of migration, respectively, with and without treatment of Rho activator CN03. Composite fluorescence microscopic images of 3D projections showing F‐actin (cyan) and nuclei (magenta). Quantification of sprout multicellularity calculated as percentage of nuclei in collective (six or more nuclei) sprouts connected to the parent vessel, relative to the total number of nuclei inside the hydrogel (*n* = 3 independent experiments, scale bar = 100 µm). All data are presented as a mean ± s.d., statistical significance was determined from a *p* < 0.05 (two‐tailed unpaired Student's *t*‐test).

It is well established that cells binding to more crosslinked, stiffer matrices encounter larger mechanical feedback upon integrin engagement. This results in more pronounced focal adhesions, F‐actin fibers, and ultimately increased RhoA/ROCK‐mediated cellular contractility, which impacts many basic cellular functions.^[^ ^13^ ^]^ We therefore speculated that changes in actomyosin‐mediated contractility could impact cell–cell adhesions, triggering the switch to single‐cell migration in highly crosslinked matrices. To test this hypothesis, we lowered cellular contractility by either pharmacologically inhibiting ROCK through Y27632 or inhibiting non‐muscle myosin II through blebbistatin, and, indeed, rescued collective migration in stiff matrices; sprouts in intermediate‐stiffness controls remained multicellular (**Figure** **2**a–d). Conversely, in matrices of intermediate crosslinking, increasing contractility by activating RhoA through CN03 induced a switch from collective to single‐cell migration (Figure 2e). In stiff hydrogels, cells treated with CN03 continued to migrate as single cells (Figure 2f). Together, these results demonstrate that more crosslinked matrices trigger ECs to migrate as single cells by upregulating their actomyosin‐based contractility.

**Figure 3:**
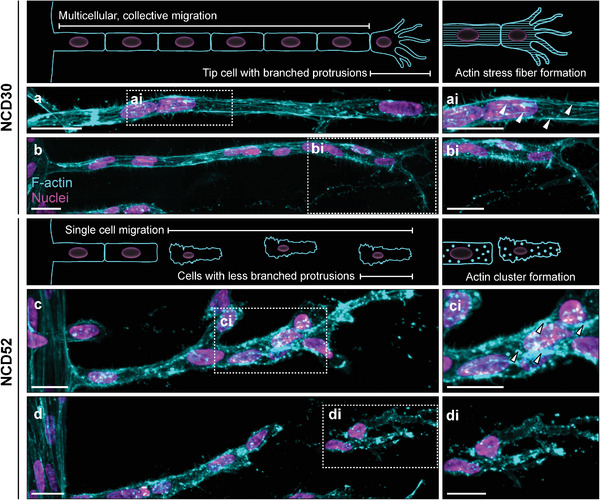
Matrix‐induced switch in EC multicellularity is accompanied by F‐actin remodeling from stress fibers to pronounced clusters. a–bi) Stress fibers and branched protrusions in a collective strand of HUVECs invading a DexMA hydrogel crosslinked with 30 × 10^−3^ m NCD peptide, fixed after 3 days of invasion. Arrowheads indicate F‐actin stress fibers. c–di) F‐actin clusters and reduced protrusions in disengaging HUVECs invading a DexMA hydrogel crosslinked with 52 × 10^−3^ m NCD peptide, fixed after 10 days of invasion. Arrowheads indicate F‐actin clusters. Composite fluorescence microscopic images of 3D projections showing F‐actin (cyan) and nuclei (magenta); scalebar = 25 µm. Supporting schematics illustrate the observed phenotypes.

Since higher contractility is generally associated with more pronounced actin stress fibers, we next characterized the actin cytoskeleton by high‐magnification imaging. In intermediately stiff hydrogels, ECs in collective strands displayed pronounced stress fibers that were oriented in the direction of migration (**Figure** **3**a,ai). Additionally, in line with previous reports,^[^ ^14^ ^]^ tip cells possessed branched, F‐actin‐rich protrusions resembling filopodia whose function is to probe and degrade the surrounding matrix (Figure 3b,bi). However, to our surprise, we found that in stiff hydrogels, the single‐cell migration phenotype was accompanied by a complete remodeling of the actin cytoskeleton, where stress fibers were no longer visible, and, instead, large punctate F‐actin clusters had formed along the entire cell cortex (Figure 3c,ci). Furthermore, compared to cells in intermediate‐stiffness hydrogels, the extent of protrusions in stiff hydrogels was drastically decreased, and the ones that formed were less branched and often displayed blunt ends (Figure 3d,di). The observed remodeling is in direct contrast to a large body of literature demonstrating that pronounced stress fibers are present on stiffer matrices,^[^ ^12^, ^15^ ^]^ giving us a first indication that the observed single‐cell migration phenotype may not primarily result from the increased mechanical feedback of the highly crosslinked, stiff matrices.

Adaptations in migration phenotypes along with cytoskeletal remodeling as a response to changes in the structural properties of 3D cellular microenvironments have previously been described for other cell types.^[^ ^16^ ^]^ Specifically, the dissolution of stress fibers and increased levels of RhoA/ROCK activity are reminiscent of the contractile amoeboid migration mode that cancer cells are able to adopt in spatially restricted environments.^[^ ^17^ ^]^ In our 3D synthetic hydrogels, the level of restriction is impacted by the density of crosslinks, which determines how fast enzymatic degradation yields the space required for migration. In other words, changes in crosslink density not only impact hydrogel stiffness but also its degradability, namely the rate at which cells can solubilize a given volume of hydrogel. Therefore, we next wanted to know whether the spatial restriction of cells, through lower degradability, was responsible for the single‐cell migration phenotype in stiff hydrogels.

**Figure 4:**
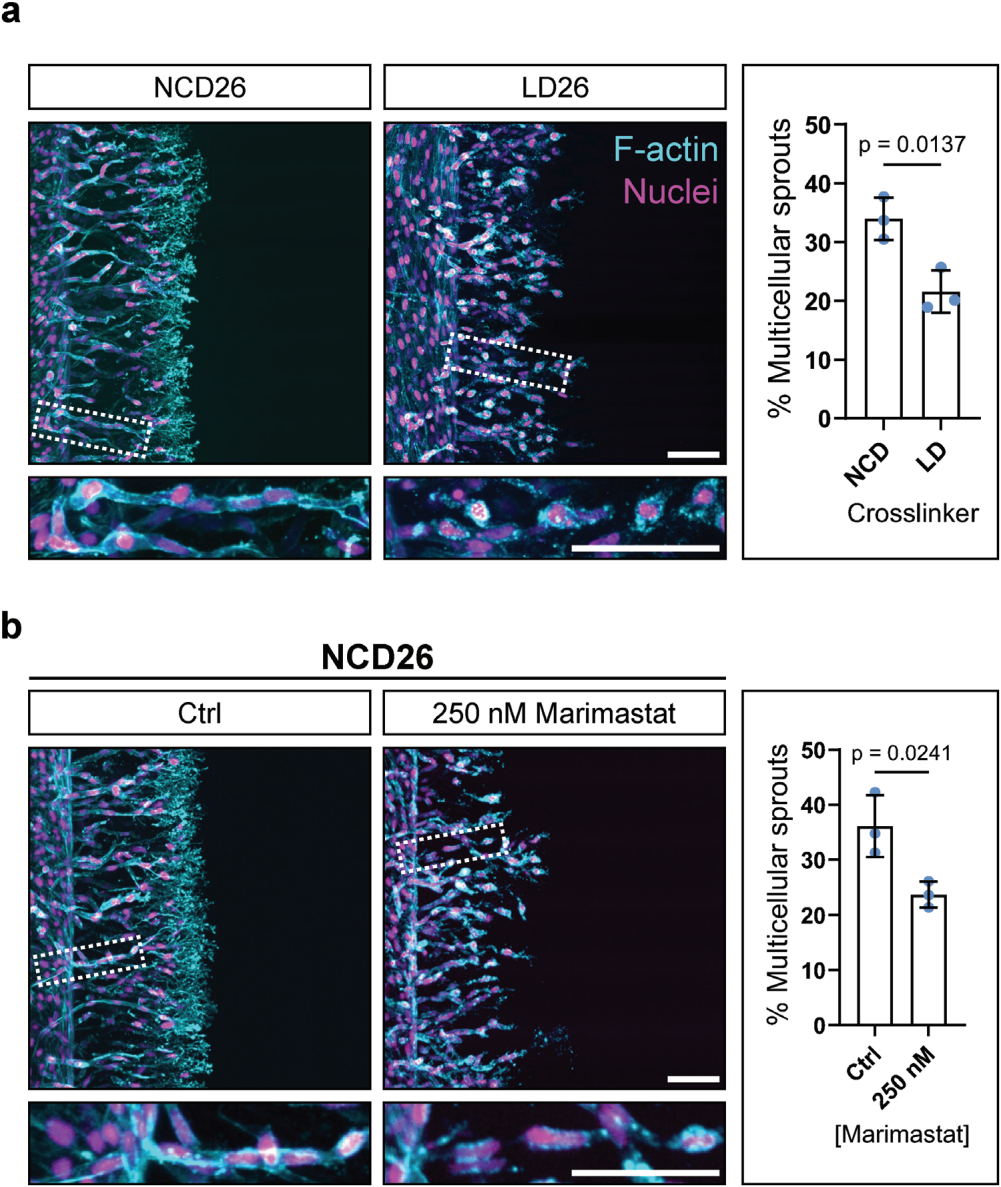
Low crosslink degradability and MMP inhibition induce single‐cell migration. a) HUVECs migrating through DexVS hydrogels crosslinked with 26 × 10^−3^ m NCD peptide or 26 × 10^−3^ m LD:NCD (1.5:1) peptide mix, fixed after 2 and 14 days of migration (at similar invasion depths), respectively. b) HUVECs migrating in DexVS hydrogels crosslinked with 26 × 10^−3^ m NCD peptide in the absence or presence of 2.5 × 10^−7^ m broad‐spectrum MMP inhibitor Marimastat. Cells were fixed after 2 or 14 days of migration, respectively. Composite fluorescence microscopic images of 3D projections showing F‐actin (cyan) and nuclei (magenta). Quantification of sprout multicellularity calculated as percentage of nuclei in collective (four or more nuclei) sprouts connected to the parent vessel, relative to the total number of nuclei inside the hydrogel (*n* = 3 independent experiments, scale bar = 100 µm). All data are presented as mean ± s.d., *p* < 0.05 is considered to be statistically significant (two‐tailed unpaired Student's *t*‐test).

In order to single out the role of matrix degradability for the observed sprouting phenotype, we maintained hydrogel crosslinking/stiffness constant (at 1.3 kPa) and modulated the susceptibility of the crosslinker peptide to cell‐secreted MMPs (Figure S1, Supporting Information). Specifically, we lowered matrix degradability by introducing an amino acid mismatch to our standard NCD crosslinker sequence derived from the cleavage site of natural type I collagen, resulting in a peptide sequence with lower MMP binding affinity (termed LD for “lower degradability” sequence).^[^ ^7d^ ^]^ Indeed, the speed of cells migrating through LD hydrogels was reduced by a factor of 8 compared to NCD controls (Figure S4a,b, Supporting Information), demanding prolonged culture periods. Since we previously observed hydrogel bulk softening with extended culture times as a result of hydrolysis in aqueous cell culture media, we replaced the hydrolysis‐prone methacrylate groups (MA) with hydrolytically stable vinyl sulfone groups (VS)^[^ ^18^ ^]^ without impacting the sprouting phenotype (Figure S5, Supporting Information). Importantly, lowering matrix degradability induced the same switch to single‐cell migration that we observed in highly crosslinked, stiff hydrogels (**Figure** **4**a), suggesting that the mechanism underlying the collectivity of invasion is controlled by matrix degradability rather than stiffness. Cells sprouting through matrices of low degradability encounter a high resistance to proteolytic cleavage, slowing down their spreading and migration, in turn triggering them to seek an alternative migration strategy.

To further support the proposed mechanism, we next confirmed that lower MMP‐mediated matrix degradation was responsible for the switch to single‐cell migration. Instead of modulating the susceptibility of the MMP‐cleavable crosslinker, we toned down MMP activity in cells migrating through more degradable hydrogels that generally support collective EC migration. We hypothesized that cells with lower MMP activity would experience high matrix resistance, even in environments that are usually optimal for cleavage. To confirm this hypothesis, we treated cells with the broad‐spectrum MMP inhibitor Marimastat, thereby reducing the cell migration speed. After treatment, cells indeed migrated individually through NCD hydrogels of intermediate crosslinking (Figure 4b), similar to the single‐cell migration pattern observed in highly crosslinked matrices. Taken together, our experiments suggest that the resistance ECs encounter upon migration through the matrix regulates the phenotype of migration. This resistance is impacted by an interplay between matrix degradability (a function of the degree of matrix crosslinking and the susceptibility of the crosslinks to MMP activity) and the activity of MMPs.

**Figure 5:**
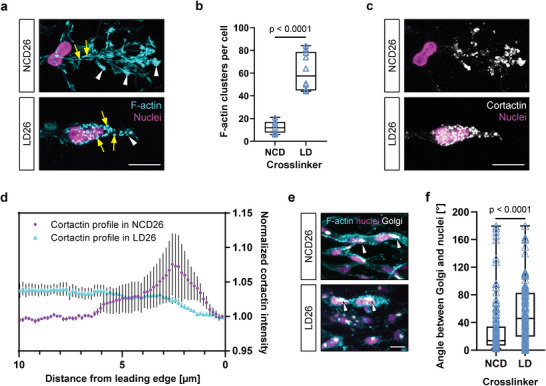
Single ECs exhibit F‐actin clusters, plain protrusions and reduced polarity in highly resistant LD matrices. a) Morphology of HUVECs migrating through a DexVS hydrogel crosslinked with 26 × 10^−3^ m NCD peptide or 26 × 10^−3^ m LD:NCD (1.5:1) peptide mix, fixed after 2 and 14 days of migration (at similar invasion depths), respectively. Yellow arrows indicate F‐actin redistribution from stress fibers (top left) to punctate clusters (bottom left). White arrowheads indicate presence (top) and absence (bottom) of branched protrusions. Composite fluorescence microscopic images showing F‐actin (cyan) and nuclei (magenta) b) Quantification of F‐actin clusters per single cell in the middle of sprouts. Clusters were counted using the Cell Counter plugin for ImageJ (manual counting, *n* = 10 cells). Data are presented as mean ± s.d. c) Morphology of HUVECs migrating through DexVS hydrogels of different crosslinker degradabilities, fixed after 2 and 14 days of migration, respectively. Cortactin localizes to the cell front in hydrogels crosslinked with 26 × 10^−3^ m NCD peptide (top), or into aggregates along the cell cortex in hydrogels crosslinked with 26 × 10^−3^ m LD:NCD (1.5:1) peptide mix. Composite fluorescence microscopic images showing cortactin (white) and nuclei (magenta). d) Cortactin intensity measured starting from the leading edge (0 µm) toward the cell center. Intensity profiles were averaged from ten cells, with three measurements per cell. Data normalized by first data point (leading edge). Error bars show means ±  SEM. e) Positioning of Golgi apparatuses in collective (top) and single HUVECs (bottom). Cells were allowed to invade a DexVS hydrogel crosslinked with 26 × 10^−3^ m NCD peptide or 26 × 10^−3^ m LD:NCD (1.5:1) peptide mix and fixed after 2 and 14 days of migration, respectively. Composite fluorescence microscopic images of 3D projections showing F‐actin (cyan), nuclei (magenta), and Golgi (white). White arrowheads indicate position of the Golgi relative to the nucleus. f) Angle analysis between nuclei and Golgi using the angle tool of ImageJ (*n* ≥ 140 cells). Data are presented as mean ± s.d. Scale bars: 20 µm. Statistical significance was determined from a *p* < 0.05 (two‐tailed unpaired Student's *t*‐test).

We next characterized the observed cellular phenotype in more detail to allow a more comprehensive classification of its hallmarks. We found that individually migrating ECs in LD hydrogels of high resistance were characterized by a reorganized actin cytoskeleton, in which stress fibers were no longer visible and F‐actin was mainly localized to cortical, punctate clusters (**Figure** **5**a,b). Another important characteristic of migrating cells is their polarity. For example, while the actin‐binding protein cortactin is usually targeted to the leading edge of protrusion‐rich cells,^[^ ^16a^ ^]^ we found that this localization differed between the two migration modes. While in NCD hydrogels of intermediate crosslinking cortactin was located at the leading edge of cells with extensive protrusions, in LD hydrogels, in which cells had switched to single‐cell migration, cortactin was redistributed to the entire cell cortex (Figure 5c,d). Moreover, this redistribution was characterized by an accumulation of cortactin in clusters, as observed for F‐actin. In addition to assessing cell polarity via cortactin distribution, we also studied polarity by investigating the localization of the Golgi apparatus relative to the nucleus. For the majority of cells migrating as collective strands through NCD hydrogels, the Golgi was positioned in front of the nucleus in the direction of migration; however, in cells migrating through LD matrices, the location of the Golgi was more random, indicating these cells’ loss of polarity (Figure 5e,f). Together, these studies highlight the decisive role of matrix degradability for ECs to switch between collective and single‐cell migration patterns, the latter being characterized by defined hallmarks commonly associated with actomyosin‐based migration strategies.

## 3 Discussion

Many studies have demonstrated the importance of the ECM in regulating cellular migration in 2D. Specifically, matrix stiffness^[^ ^19^ ^]^ and adhesiveness^[^ ^20^ ^]^ have emerged as critical parameters that affect the phenotype of migrating cells. However, how individual matrix properties impact cell migration in more physiological 3D matrices remains poorly understood. In contrast to cell migration on surfaces, cells embedded in 3D tissues can only migrate if they overcome the restricting ECM barrier by physical or proteolytic remodeling.^[^ ^4^ ^]^ Depending on ECM composition, which varies greatly between different tissue types,^[^ ^21^ ^]^ this barrier can be more or less resistant to cellular remodeling; how the degree of ECM resistance impacts cell migration is not known.

Recent work has shown that the level of spatial confinement can regulate the collectivity of migrating cancer cells.^[^ ^5^ ^]^ However, these studies were performed in 3D materials of natural origin (such as type I collagen hydrogels or decellularized tissues), in which it is difficult to control individual matrix parameters independently in order to unambiguously determine their individual regulatory role. For example, in 3D collagen hydrogels, matrix stiffness is commonly controlled by tuning the protein content.^[^ ^5^, ^22^ ^]^ However, these modifications also alter collagen fiber thickness, pore size of the meshwork, hydrogel degradability and cell‐adhesive ligand concentration or availability, all of which are known to regulate many important cell functions.^[^ ^10^ ^]^ Due to these limitations, a full understanding of how individual structural ECM cues control cellular migration patterns is still lacking. For example, some studies using natural type I collagen hydrogels have suggested that single‐cell migration is favored in large pore‐size environments, whereas small pore‐size matrices support multicellular migration.^[^ ^5a^ ^]^ In contrast, when cells migrated through narrow microchannels with adjustable widths, single‐cell migration was favored in smaller channel diameters as opposed to larger diameters.^[^ ^23^ ^]^ Resolving this discrepancy requires access to experimental models that allow for independent tuning over physical resistance in a 3D environment that mimics physiological tissues. Here, using a synthetic hydrogel with independent control over matrix properties, we were able to uncover the impact of matrix resistance on EC migration in the absence of changes in confounding parameters, including adhesive ligand density or pore size.

While ECM‐dependent switches in migration modes have been well characterized for several cell types, such as fibroblasts and cancer cells,^[^ ^5^, ^16^, ^24^ ^]^ it is not at all known whether similar microenvironmental control mechanisms exist for other cell types. In particular, ECs have to cleave dense 3D tissues in order to migrate and form new blood vessels; whether and how the matrix regulates EC migration is poorly understood. Some studies have suggested a switch to amoeboid phenotypes when the expression of MMPs is downregulated, which could be an indication of a potential matrix control.^[^ ^25^ ^]^ We have previously shown that changes in hydrogel crosslinking density cause ECs to switch between collective strands at intermediate crosslinking levels and single cells at high and low crosslinking levels. While we determined that the reason for the disengagement of cell–cell contacts in lightly crosslinked hydrogels was the high matrix degradability resulting in fast EC migration,^[^ ^7d^ ^]^ it remained unclear why highly crosslinked matrices also resulted in single‐cell migration.

**Figure 6:**
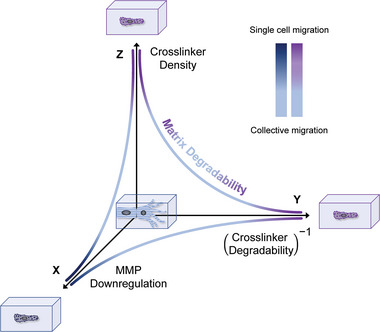
Model of matrix resistance controlling multicellularity of EC migration in 3D. The migration mode of ECs is regulated by the resistance of 3D matrices to proteolytic cleavage. The overall level of resistance cells experience is a function of the crosslinker concentration (Z) as well as its degradability (Y), and is relative to the cell secretion rate of MMPs (X). When matrix resistance is low, multicellular migration is supported. In turn, high matrix resistance induces single‐cell migration. The origin is defined as intermediate crosslinking density, NCD crosslinker and non‐perturbed MMP activity. Color codes indicate collective migration (light blue), or single‐cell migration induced by the specific MMP activity (dark blue) or matrix properties (purple).

In the current study, we found that highly crosslinked matrices present an environment to cells that largely resists enzymatic cleavage, thereby hindering their migration. The overall level of matrix resistance is determined by, on the one hand, the number of crosslinks, and, on the other, the crosslinker's degradability. Whether a cell senses a given matrix as resistant or not depends on the expression and activity of cellular MMPs, which vary between cell types^[^ ^26^ ^]^ and different microenvironmental contexts.^[^ ^27^ ^]^ Based on our findings, we developed a model (**Figure** **6**) demonstrating how these factors relate: In this theoretical diagram, we report the analyzed matrix and cellular properties on three axes: crosslinker density (*Z* axis), crosslinker degradability (*Y* axis) expressed as a reciprocal, and MMP activity (*X* axis). The origin of this multi‐dimensional space is defined as our standard condition, characterized by low levels of matrix resistance and collective EC migration. Any movement along the axes results in increased matrix resistance, promoting a switch to single EC migration. We speculate that cells sense resistant matrices as a physical barrier that is too difficult to be cleaved by secreted MMPs, in turn inhibiting their spreading and protrusion formation. This speculation is in line with previous studies by us and others reporting delayed spreading kinetics when cells are embedded in highly crosslinked matrices that are not easily degradable.^[^ ^28^ ^]^ As a result, cells are unable to adopt the elongated shape that is required for strand‐like migration and, instead, seek an alternative migratory strategy that does not depend on MMP cleavage.^[^ ^16^, ^24^ ^]^ In natural hydrogels with relatively large pores similar to the size of cells, such migration mechanisms can be an efficient way to move forward^[^ ^16b^ ^]^; however, our nanoporous gels depict a physical barrier that cells cannot pass through without degrading it. Indeed, we observed that cells ultimately stop migrating in high‐resistance hydrogels (Figure S4, Supporting Information), which is a further indication that they have indeed switched to an MMP‐independent mode. Taken together, our data show that ECs, similar to cancer cells,^[^ ^5^ ^]^ adjust their migration strategy depending on the physical properties of the microenvironment.

The single‐cell migration of ECs in high‐resistance environments shares cellular characteristics with other described ECM‐dependent migration mode switches. For example, some hallmarks are shared with the amoeboid migration mode that cancer cells are able to adopt: a clear Rho/ROCK and actomyosin dependency, a reduction of branched protrusions and severe cytoskeletal remodeling.^[^ ^17^ ^]^ Additionally, fibroblasts can undergo a matrix‐induced switch to intracellular pressure‐driven lobopodial migration, in which cells use the nucleus as a piston to physically push through 3D matrices.^[^ ^16a^ ^]^ Many hallmarks described for lobopodial cells (high contractility state of cells, cortactin reorganization, partial loss of cell polarity and MMP‐independent locomotion) match the characteristics of ECs described here. However, one major difference is the presence of stress fibers in lobopodial cells, distinguishing them from single ECs in resistant matrices.^[^ ^29^ ^]^ In the future, further characterization of the underlying cellular signaling mechanisms will be required to gain a full understanding of how the physical matrix properties are transduced intracellularly. Furthermore, in vivo studies will be needed to validate the findings in living tissues and to probe whether the matrix‐induced changes in EC migration patterns are linked to pathophysiological disease phenotypes in which matrix remodeling plays a role.

Overall, we define matrix resistance as a previously unknown microenvironmental cue that toggles ECs and potentially other cell types between collective and single‐cell migration modes characterized by distinct cytoskeletal states, thereby contributing to a better understanding of how physical ECM properties regulate complex cellular functions. Given the importance of angiogenic sprouting for tissue homeostasis and disease, our findings not only provide a previously unknown matrix cue that regulates cell migration patterns in 3D, but they also contribute to a better mechanistic understanding of some of the most fundamental processes of microvessel formation.

## 4 Experimental Section

### Reagents

All reagents were purchased from Sigma–Aldrich, unless stated otherwise.

### Peptides and Cell Adhesive Ligands

Cell adhesive ligand CGRGDS as well as crosslinker peptides of native collagen degradability CGPQGIAGQGCR (NCD‐CR), KCGPQGIAGQCK (NCD‐KK) and low degradability CGPQGPAGQGCR (LD) were custom synthesized by GenScript and provided as hydrochloride salts (purity > 95%).

### Antibodies

Rabbit antihuman cortactin antibody (H222) (1:200) was purchased from Cell Signaling (#3503), rabbit antihuman giantin antibody (Poly19243) (1:000) was purchased from BioLegend (#924302). Secondary antibody Alexa Fluor 647 goat anti‐rabbit IgG (1:1000) was purchased from Thermo Fisher (#A‐21244).

### Synthesis of DexMA

Methacrylated dextran was synthesized as previously described.^[^ ^7^, ^30^ ^]^ In brief, dextran (20 g, MP Biomedicals, MW 86 000 Da) and 4‐dimethylaminopyridine (2 g) were dissolved in 100 mL anhydrous dimethyl sulfoxide. Glycidyl methacrylate (24.6 mL) was added under stirring, the mixture heated to 45 °C, and the reaction allowed to proceed for 24 h. The solution was then precipitated into cold 2‐propanol (1 L, VWR) and the crude product was collected. Next, the crude product was re‐solubilized in Milli‐Q water and dialyzed against Milli‐Q water for 3 days. The final product was obtained by lyophilization. A methacrylate/dextran repeat unit ratio of 0.7 was determined by ^1^H NMR spectroscopy.

### Synthesis of DexVS

Vinyl sulfone functionalized dextran was prepared as previously reported.^[^ ^30^, ^31^ ^]^ In brief, divinyl sulfone (4.96 mL) was added to a solution of dextran (4 g, MP Biomedicals, MW 86,000 Da) in aqueous sodium hydroxide (0.1 m, 100 mL) under vigorous stirring at room temperature. The reaction was stopped after 5 min by adding hydrochloric acid to adjust the pH of the solution to 5. Purification of the crude product was achieved through dialysis (SnakeSkin Dialysis Tubing, Life Technologies, 10 kDa cutoff) against Milli‐Q water for 3 days with two water changes daily. The final product was recovered by lyophilization. A vinyl sulfone/dextran repeat unit ratio of 0.5 was determined by ^1^H NMR spectroscopy.

### Preparation of MMP‐Cleavable DexMA Hydrogels

DexMA hydrogels crosslinked with the MMP cleavable peptide NCD‐CR were used for all crosslinker density studies in Figures 1, 2, 3 and prepared as previously described.^[^ ^30b^ ^]^ Briefly, solutions of DexMA (4.4% w/v) and CGRGDS (final concentration 6 × 10^−3^ m) were prepared in Dulbecco Modified Eagle Medium (DMEM, pH 7.0). The pH was adjusted to 8.0 to initiate the coupling of the adhesive ligand to DexMA. After 30 min of reaction, NCD‐CR crosslinker solution in DMEM, pH 7.0, was added to the reaction mixture at final concentrations of 30.5 × 10^−3^ or 52 × 10^−3^ m, respectively, and the pH readjusted to 8.0 to initiate hydrogel formation. The solution was immediately pipetted into the main chamber of the microfluidic device and allowed to crosslink in a humid atmosphere for 30 min. The device was covered with phosphate‐buffered‐saline (PBS) and kept in a cell culture incubator with constant humidity at 37 °C and 5% CO_2_.

### Preparation of MMP‐Cleavable DexVS Hydrogels

Due to their increased hydrolytic stability, MMP‐cleavable DexVS hydrogels were used for all LD experiments in Figures 4 and 5 requiring long culture periods. DexVS hydrogels were prepared as described previously.^[^ ^30b^ ^]^ In brief, a neutralized solution of CGRGDS (final concentration 6 × 10^−3^ m) was prepared in PBS/phenol red and reacted with DexVS (final concentration of 4.2% w/v) on ice. Coupling was initiated by adjusting the pH to 7.5 (using NaOH, 0.2 m) and proceeded at room temperature for 30 min. Next, control samples were crosslinked with NCD‐KK peptide and LD samples with a mix of LD and NCD‐KK peptide at a ratio of 1.5:1. To initiate coupling, peptide crosslinker solution in PBS/phenol red was added to the reaction mixture at a final concentration of 25.2 × 10^−3^ m. The precursor solution was cooled down on ice and neutralized with NaOH (0.2 m) to pH 7.5 to initiate hydrogel crosslinking. Immediately, the hydrogel was added to the central hydrogel chamber. Finally, the hydrogel was allowed to fully polymerize by incubating for another 30 min at room temperature in moist atmosphere. The device was covered with PBS and kept in a cell culture incubator with constant humidity at 37 °C and 5% CO_2_.

### Mechanical Testing of Hydrogels

Young's moduli of DexMA and DexVS hydrogels were characterized using a nanoindenter (Piuma, Optics 11, Netherlands). A cantilever with a spring constant of 0.026 N m^−1^ and bead diameter of 23.5 µm was used. The Young's modulus of each hydrogel was averaged from at least ten indentations on three independent hydrogels of 6 mm diameter immersed in PBS supplemented with 2% FBS, see Figure S1 (Supporting Information). Indentation curves were fitted to the Hertzian contact model.

### Cell Culture

HUVECs were obtained from Lonza (#C2519A). They were cultured on 0.1% gelatin coated dishes in fully supplemented EGM‐2 medium (PromoCell) with an additional 250 ng mL^−1^ amphotericin B and 10 µg mL^−1^ gentamicin (Gibco). Cell cultures were kept in incubators with constant humidity at 37 °C and 5% CO_2_. Cells at passage 5 were used in all angiogenic sprouting assays.

### Angiogenic Device Fabrication

Microfluidic devices for in vitro angiogenic sprouting studies were fabricated as previously published. In brief, the device housing consisted of two patterned PDMS layers molded from photolitographically generated silicon masters, which were bonded to each other and sealed against a glass coverslip. To embed cylindrical channels into the hydrogel, two acupuncture needles (Hwato, Ø = 400 µm) were coated with 5% w/v aqueous gelatin solution, cooled to 4 °C and inserted into a UV‐sterilized device. The hydrogel precursor solution was cast into the central device chamber and allowed to polymerize for 30 min at room temperature. The resulting hydrogels were equilibrated in PBS overnight at 37 °C to melt the gelatin coating. Needle extraction was followed by thorough washing with PBS and EGM‐2 prior to cell seeding.

### Angiogenic Sprouting Assays in Dextran Hydrogels

For angiogenic sprouting experiments, HUVECs were seeded through one reservoir at 10 million cells mL^−1^ EGM‐2 and were allowed to adhere to the bottom surface of the channel for 30 min, followed by cell seeding of the top channel side for an additional 30 min, achieved by device inversion. Cells that adhered to the reservoirs were scratched off and removed by medium exchange. Devices were kept on a platform rocker (BenchRocker BR2000) to establish gravity‐driven flow through both channels. 6 h after seeding, a chemokine cocktail consisting of 75 ng mL^−1^ vascular endothelial growth factor (rhVEGF 165, R&D Systems), 150 ng mL^−1^ PMA (Sigma) and 2.5 × 10^−7^ m sphingosine‐1‐phosphate (S1P, Cayman Chemical) in culture medium was applied to the second, parallel channel to induce angiogenic sprouting. Composition of the chemokine cocktail was taken from a previously published recipe, in which ingredients and concentrations had been titrated to support multicellular sprouting in DexMA hydrogels of intermediate crosslinking density.^[^ ^7d^ ^]^ Agents for RhoA‐ROCK‐myosin II studies Rho Activator II (#CN03, Cytoskeleton), Y27632 (#203389, Sigma–Aldrich) and Blebbistatin (#203389, Sigma–Aldrich) were administered into both channels at 5 µg mL^−1^, 10 × 10^−6^ m and 10 × 10^−6^ m respectively. MMP inhibitor Marimastat (Tocris Bioscience) was added to both channels at 2.5 × 10^−7^ m.

### Rhodamine B and FITC‐VEGF‐A_165_ Diffusion

S1P (379.47 g mol^−1^) and PMA (616.83 g mol^−1^) diffusion in DexMA hydrogel was modeled by the fluorescent dye Rhodamine B (479.02 g mol^−1^) due to its similar molecular weight. Rhodamine B (5 µg mL^−1^ in PBS) was added to one channel of the microfluidic device and allowed to diffuse for 30 min through DexMA hydrogels crosslinked with 52 × 10^−3^ m NCD peptide, the other channel was filled with PBS. The fluorescence signal was acquired, and the procedure repeated every 24 h. Rhodamine B and PBS were exchanged daily 30 min prior to image acquisition. Throughout the experiment, devices were kept on a platform rocker at 37 °C and 5% CO_2_.

Diffusion of human VEGF‐A_165_ was visualized by FITC‐labeled recombinant human VEGF‐A_165_. Protein labeling and purification was performed using FluoReporter FITC Protein Labeling Kit (Invitrogen) following the manufacturer's instructions. Briefly, recombinant human VEGF‐A_165_ (Peprotech) was reacted with reactive dye (100 molar excess relative to protein monomer) in 0.1 m NaHCO_3_ buffer (pH 9.0). Reaction was performed for 1 h at room temperature in the dark, followed by purification via size‐exclusion chromatography through spin columns provided by the labeling kit. Assuming 85% recovery of labeled‐protein, a 0.5 degree of labeling (dye per protein molecule) was estimated. FITC‐labeled recombinant human VEGF‐A_165_, stored in 1% BSA solution at −80 °C, was added to the microfluidic device at a concentration of 5 µg mL^−1^ in EGM‐2 and handled as described above. The fluorescence signal was acquired, followed by exchange of VEGF‐A_165_ and EGM‐2, every 24 h.

### Fluorescent Staining, Microscopy and Analysis

HUVECs in devices were fixed with 4% paraformaldehyde solution in PBS (Thermo Fisher) for 1 h at room temperature, followed by cell permeabilization and blocking with 0.5% Triton X100 and 3% BSA in PBS for 1 h at room temperature. For antibody staining, samples were incubated in primary antibody diluted in 3% BSA at 4 °C for 24 h. Devices were then soaked in 0.1% Tween in PBS at 4 °C for 2 days with multiple buffer exchanges to wash out excess antibody. Next, samples were incubated with Alexa Fluor 647‐conjugated secondary antibody overnight at 4 °C and washed for 1 day with 0.1% Tween. Finally, cell nuclei were stained with Hoechst 33342 (1:200, Life Technologies) and F‐actin with Alexa Fluor 488 Phalloidin (1:500, Thermo Fisher) overnight at 4 °C. This step was applied to all samples, independent of an additional antibody staining. All incubation steps were performed on a platform rocker to improve the diffusion of antibodies, dyes and washing agents into the 3D hydrogels.

Fixed and stained samples were imaged at 10× and 20× (sprout overview) or 40×, 60×, and 80× (single sprouts and single cells) using a confocal spinning disc microscope (Dragonfly by Andor with dedicated operation software Fusion, v2.0.0.13). Microscopic fluorescence images are presented as maximum intensity projections. Sprout multicellularity was determined via nuclei detection (spot assistant) in IMARIS (vx64, 9.1.1). The degree of multicellularity was analyzed by manually counting the number of nuclei per sprout connected to the parent vessel. A subset of spots was created per sprout. Sprouts with at least six nuclei (crosslinker density studies, 400 µm migration distance) or four nuclei (LD studies, ≈250 µm maximum migration distance) were considered multicellular. The number of nuclei in multicellular sprouts was presented relative to the total number of nuclei. Cortactin intensity profiles were acquired from the leading edge (0 µm) toward the cell center and averaged from 10 cells, with three measurements per cell. Data were normalized by the first data point (at the leading edge). To quantify the number of actin clusters per cell, the Cell Counter (v3.0.0) plugin for ImageJ (manual counting, *n* = 10) was used. Rhodamine B and FITC‐VEGF intensity profiles were acquired from the edge of the source channel (0 µm) toward the edge of the other, parallel channel and averaged over five measurements per image. Data were normalized to the highest intensity value acquired at day 3. Scatter plots were generated with GraphPad Prism 9 (v9.5.0). Brightfield images were acquired with a Leica DMi1 inverted microscope (with built‐in software Leica application suite, v3.4.0).

### Statistics

All statistical analyses were carried out using GraphPad Prism 9 (v9.5.0). Statistical significance was determined by two‐tailed unpaired Student's *t*‐test. *p* values < 0.05 were considered statistically significant. All data are presented as a mean ± standard deviation unless stated otherwise. Each study was independently repeated three times or as specified.

## Conflict of Interest

The authors declare no conflict of interests.

## Author Contributions

M.S.W., G.T., and B.T. conceived the study, designed experiments, and interpreted results; M.S.W. conducted experiments and analyzed data; H.L. characterized hydrogels by nanoindentation; M.S.W., G.T., and B.T. wrote the manuscript.

## Supporting information

Supporting Information

## Data Availability

The data that support the findings of this study are available from the corresponding author upon reasonable request.
