# Supplementary material for: Matrix Resistance Toward Proteolytic Cleavage Controls Contractility‐Dependent Migration Modes During Angiogenic Sprouting
Source: Adv Sci (Weinh). 2024 Mar 13;11(19):2305947. doi: 10.1002/advs.202305947 (PMC11109655; doi:10.1002/advs.202305947)
Supplement: Supplementary file 1 — Supporting Information [file ADVS-11-2305947-s001.pdf]

ADVANCED SCIENCE
Open Access

Supporting Information

for *Adv. Sci.*, DOI 10.1002/advs.202305947

Matrix Resistance Toward Proteolytic Cleavage Controls Contractility-Dependent Migration Modes During Angiogenic Sprouting

*Martin S. Weiß, Giuseppe Trapani, Hongyan Long and Britta Trappmann**

Supporting Information

Matrix resistance toward proteolytic cleavage controls contractility-dependent migration modes during angiogenic sprouting

*Martin S. Weiß, Giuseppe Trapani, Hongyan Long, Britta Trappmann**

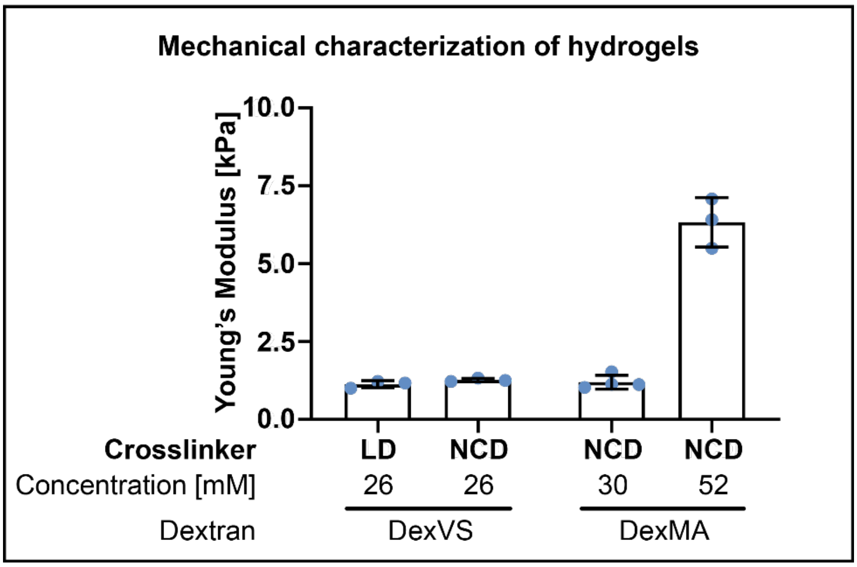

Figure S1. Mechanical characterization of dextran-based hydrogels. Young's moduli of DexMA and DexVS hydrogels crosslinked with varying concentrations of NCD peptide. LD gels were crosslinked with 26 × 10^{-3} M LD:NCD (1.5:1) peptide mix. Data are presented as mean ± s.d.

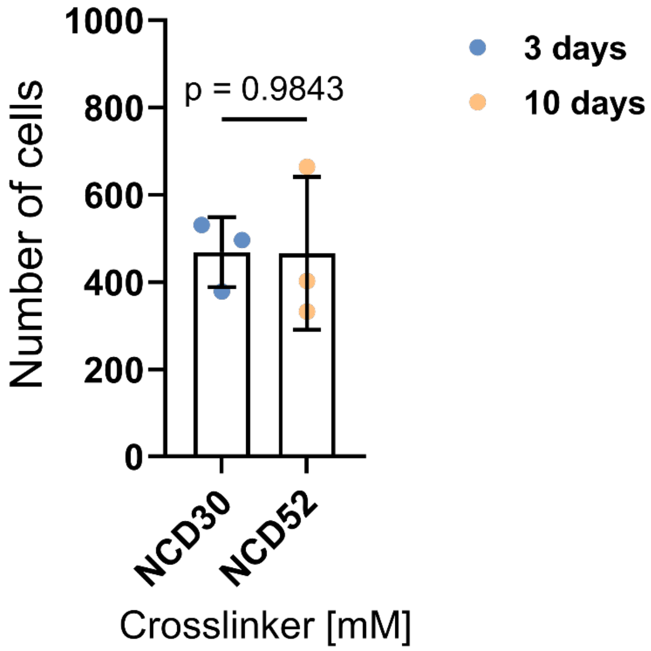

Figure S2. Total number of invaded cells depends on invasion depth, not culture time. Quantification of total cell number after 3 and 10 days of migration in DexMA hydrogels, respectively. Analyzed samples contained 30×10^{-3} M or 52×10^{-3} M NCD crosslinker peptide (n = 3 independent experiments). Samples were fixed at a constant cell invasion depth of ~400 µm. All data shown as mean ± s.d., p-values below 0.05 (two-tailed unpaired Student's t-test) were considered statistically significant.

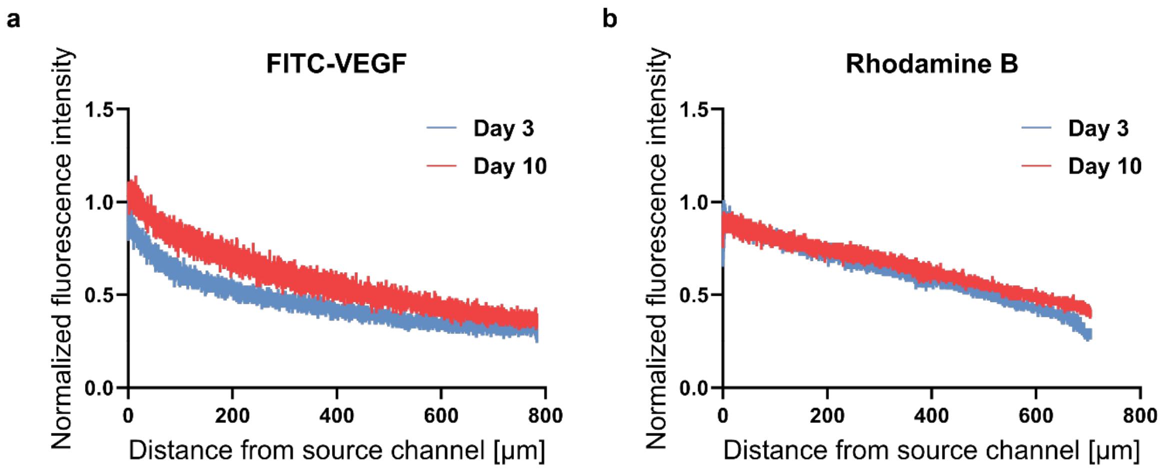

Figure S3. Pro-angiogenic cocktail components do not accumulate in synthetic hydrogels over time. (a,b), Fluorescence intensity profiles of FITC-labeled recombinant human VEGF-A_{165} (a) and Rhodamine B (b) diffusing through DexMA hydrogels crosslinked with 52 × 10^{-3} M NCD peptide, at 3 and 10 days, respectively. FITC-VEGF (5 µg/mL in EGM-2) was added to one channel of the microfluidic device and imaged after 24 h of diffusion. EGM-2 (added to the other, parallel channel) and FITC-VEGF were refreshed following image acquisition. Rhodamine B (simulating small molecules S1P and PMA, 5 µg/mL in PBS) was added to one channel of the microfluidic device and imaged after 30 minutes of diffusion. PBS (added to the other, parallel channel) and Rhodamine B were exchanged and replenished every 24 h. Fluorescence intensity was measured starting from the channel edge (0 µm) toward the edge of the second channel. Intensity profiles were averaged from five measurements per image (n = 1) and normalized to the highest intensity value acquired at day 3.

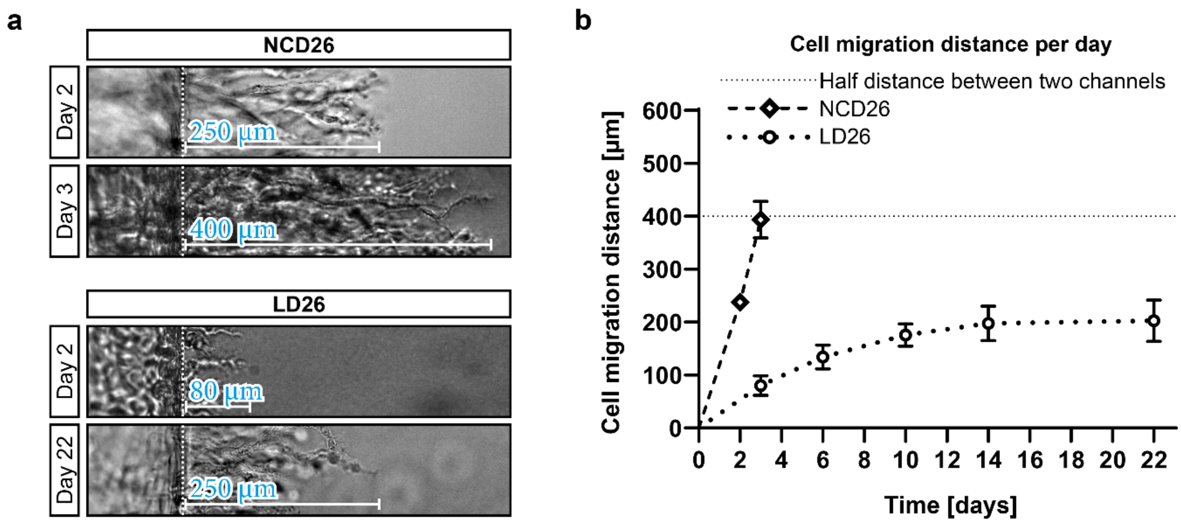

Figure S4. ECs stop migrating in LD matrices over time. (a), Brightfield images of HUVECs migrating through hydrogels crosslinked with 26 × 10^{-3} M NCD peptide (top) or 26 × 10^{-3} M LD:NCD (1.5:1) peptide mix (bottom), fixed after 3 and 22 days of invasion, respectively. Cells in LD hydrogels gradually stop migrating. (b), Quantification of cell migration distance per day. Analyzed samples contained 26 × 10^{-3} M NCD peptide or 26 × 10^{-3} M LD:NCD (1.5:1) peptide mix (n = 3 independent experiments).

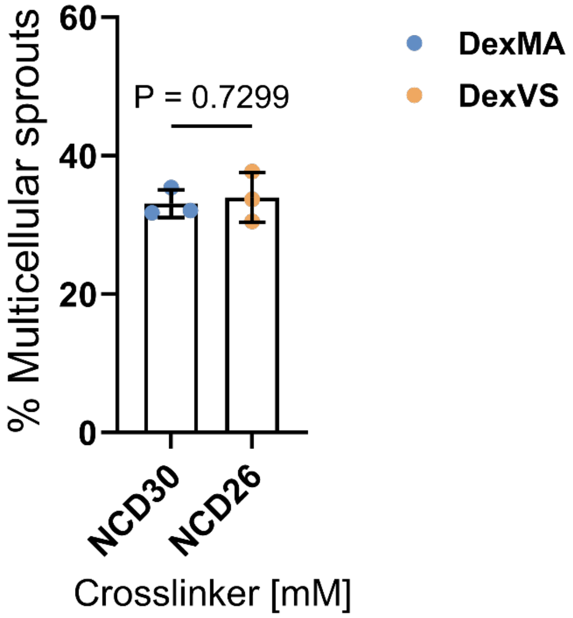

Figure S5. Multicellularity of angiogenic sprouts is not affected by different biopolymers. Quantification of HUVEC-multicellularity migrating through DexMA or DexVS hydrogels crosslinked with 30×10^{-3} M NCD peptide or 26×10^{-3} M NCD peptide, fixed after 3 days of migration, respectively. Quantification of sprout multicellularity calculated as percentage of nuclei in collective (6 or more nuclei) sprouts connected to the parent vessel, relative to the total number of nuclei inside the hydrogel (n = 3 independent experiments). All data are presented as mean ± s.d., $p < 0.05$ is considered to be statistically significant (two-tailed unpaired Student's t-test).
